# Infected cephalhaematoma in a five-week-old infant - case report and review of the literature

**DOI:** 10.1186/s12879-016-1982-4

**Published:** 2016-11-04

**Authors:** Petra Zimmermann, Andrea Duppenthaler

**Affiliations:** 1 Infectious Diseases Unit, University Children’s Hospital Berne, Freiburgstrasse 10, 3010 Berne, Switzerland; 2 Infectious Diseases Unit, The Royal Children’s Hospital Melbourne, Parkville, Australia

**Keywords:** Cephalhematoma, Infection, Management, Review, *Escherichia coli*, Neonates

## Abstract

**Background:**

A cephalhaematoma is usually a benign condition which resolves spontaneously. Nevertheless, there is a small risk of primary or secondary infection and diagnosis of this condition is challenging. The purpose of this article is to summarise risk factors, clinical criteria, pathogenesis, appropriate investigations and treatment methods for infected cephalhaematomas in infants.

**Case presentation:**

A 5-week-old infant presented with fever and a non-tender cephalhaematoma without local signs of inflammation. The inflammatory markers in blood were elevated. Urine, blood and cerebrospinal fluid cultures were sterile. The raised inflammatory markers did not decrease under antibiotic treatment. An aspirate of the cephalhaematoma grew *Escherichia coli*. A debridement and evacuation of the haematoma was performed and the infant was treated with antibiotics for 11 days. The infant did not show any sequelae on follow-up visits.

**Conclusions:**

We present a case of an infected cephalhaematoma with *Escherichia coli* in a 5-week-old infant. Diagnosis of an infected cephalhaematoma is challenging. Infection should be suspected if infant present with secondary enlargement of the haematoma, erythema, fluctuance, skin lesions or signs of systemic infection. Inflammatory markers and imaging have limited diagnostic power. The main associations with infection of cephalhaematomas are instrumental assisted deliveries and sepsis, followed by the use of scalp electrodes, skin abrasions and prolonged rupture of membranes. Although, aspiration is contraindicated in treatment of cephalhaematomas, it needs to be performed when an infection is suspected. *Escherichia coli* are the most frequently isolated bacteria from infected cephalhaematomas.

## Background

A cephaelhaematoma is defined as a subperiosteal haemorrhage in a newborn secondary to birth trauma. It occurs in 1–2 % of spontaneous vaginal deliveries and 3–4 % of forceps or vacuum-assisted deliveries [1]. Usually, it is a benign condition which resolves spontaneously over weeks without any treatment. Possible complications are hypotension, anaemia, jaundice or exostosis [2]. Moreover, there is also a potential risk of either primary infection through skin lesions or secondary infection through bacteraemia.

We present an illustrative case of an infant with an infected cephalhaematoma which was initially misdiagnosed. We added a literature review summarizing clinical presentation, risk factors, investigations results and treatment of previously reported cases.

## Case presentation

**Fig. 1:**
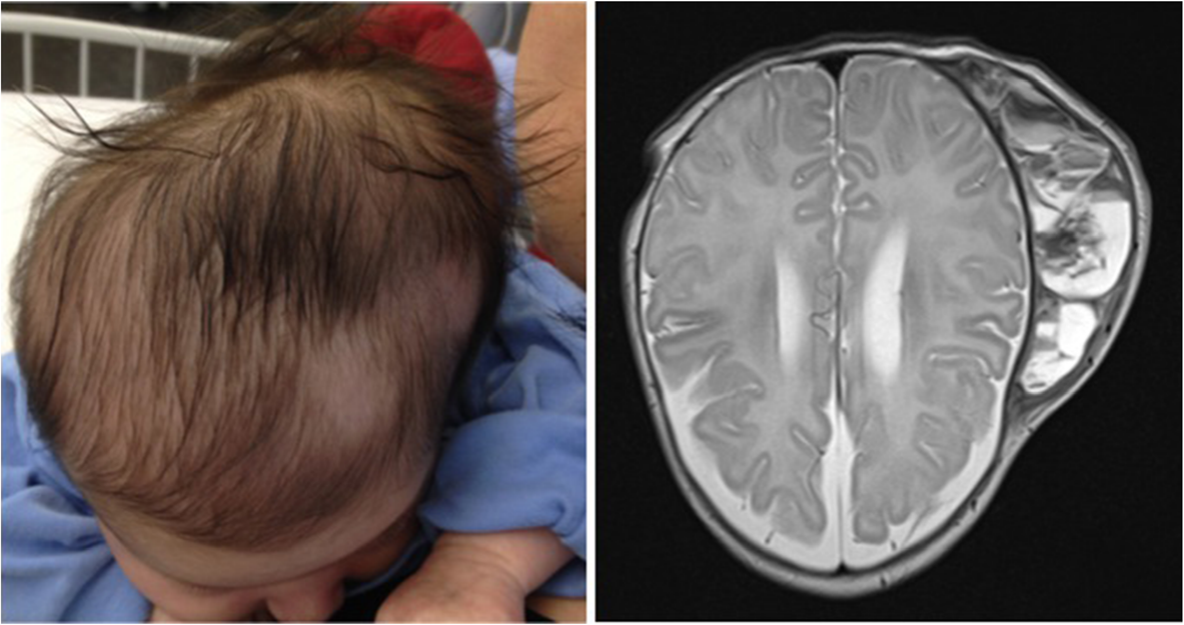
Clinical presentation and magnetic resonance imaging findings of the 5-week-old girl with infected cephalhaematoma

A 5-week-old girl presented with decreased feeding and a fever of 38.5 °C. The infant was delivered at term by vacuum-extraction after an uneventful pregnancy. There was no protracted labour or use of a scalp electrode. A cephalhaematoma on the left parieto-temporal region was noted on the first day of life. At presentation on the 35th day of life, the only clinical symptom, apart from fever, was a non-tender cephalhaematoma of 12 × 16 cm without local signs of inflammation (Fig. 1). Laboratory tests revealed a haemoglobin level of 79 g/l, a total white blood cell count of 13.2 × 10^9^/l, a platelet count of 233 × 10^9^/l, a C-reactive protein level of 212 mg/l and an erythrocyte sedimentation rate above 110 mm/h. The girl was started on intravenous treatment with ceftriaxone 95 mg/kg/d and amoxicillin/clavulanate 150 mg/kg/d. Urine, blood and cerebrospinal fluid cultures remained sterile. A magnetic resonance imaging of the whole body, which was done in order to search for an infectious focus, revealed no other pathology apart from the cephalhaematoma (Fig. 1). The elevated inflammatory markers did not decrease under antibiotic treatment and were thought to be a reaction to the resorption process of the haematoma. The antibiotic therapy was stopped after 10 days and the girl was discharged.

On follow-up one week later, a 2 × 2 cm sized, fluctuant, reddened superimposed area with blisters had formed on the cephalhaematoma. A diagnostic tap was done and the girl was started on intravenous cefuroxime 150 mg/kg/d. On the next day a debridement and evacuation of the haematoma was performed. The culture of the aspirate grew *Escherichia coli.* According to the antibiotic suspectibilities the treatment was changed to amoxicillin 150 mg/kg/d for total duration of 11 days. On follow-up four months later, the girl was well. There were no residual local findings and the neuropsychological development was normal.

## Conclusion

A literature review was performed using Medline and Web of Science (1950 to January 2016) with the search terms: (cephalhematoma OR cephalhaematoma OR cephalohematoma) AND (infected OR infection) AND (treatment OR management). References were hand-searched for additional articles. Criteria for inclusion of publications were: (i) infants less than 4 months of age, (ii) case reports supplying sufficiently detailed clinical, diagnostic and therapeutic data, (iii) publications in English, Spanish, Italian, French or German. A total of 36 publications containing 42 case reports plus our case were included in the review [1, 3–37]. Descriptive statistical analysis was performed analysing demographic data, risk factors, clinical presentation, investigation results and treatment. Neonates were classified as preterm babies when born at a gestational age of less than 37 weeks. Prolonged rupture of membranes was defined as rupture ≥ 18 h before delivery. Fever was defined as a body temperature ≥ 38 °C and leucocytosis as a total white blood cell count ≥ 15 × 10^9^/l. Infants were categorised as non-traumatic cases when delivered spontaneously without the use of a scalp electrode and in absence of skin abrasions. The time of diagnosis was defined as the time when the percutaneous tap was performed. The diagnosis of sepsis was made by positive blood cultures and of meningitis by positive cultures from cerebrospinal fluid. Osteomyelitis was either diagnosed when osteolytic lesions were present on skull X-rays or lytic bone changes on computed tomography images.

The clinical characteristics of the 43 infants with infected cephalhaematomas are summarised in (Table 1). The median age at the time of the first medical presentation was 10 (range 2–98, mean 15) days. The main reasons for presentation were an increase in size of the cephalhaematoma (*n* = 21, 49 %) or fever (*n* = 20, 47 %). Other reasons were poor feeding (*n* = 7, 16 %), lethargy (*n* = 8, 19 %), icteric skin colour (*n* = 8, 19 %) or pallor (*n* = 3, 7 %). One patient presented with seizures.

**Table 1:** Demographic and clinical characteristics of the 43 infants with infected cephalhaematomas

Characteristic	Number of patients (%)
Gender	
Female	26 (60)
Male	12 (28)
Not specified	5 (12)
Gestational age	
Term	40 (93)
Preterm^a^	3 (7)
Risk factors	
Assisted delivery	19 (44)
Scalp electrode	10 (23)
Skin abrasion	9 (21)
Prolonged rupture of membranes^b^	3 (7)
Sepsis	18 (42)
Urinary tract infection^c^	1 (2)
Location of cephalhaematoma	
Parietal	29 (67)
Parieto-occipital	8 (19)
Parieto-temporal	2 (5)
Location not specified	4 (9)
Right	18 (42)
Left	12 (28)
Bilateral	11 (25)
Side not specified	2 (5)
Reasons for presentation	
Increasing swelling	21 (49)
Fever	20 (47)
Poor feeding	7 (16)
Lethargy	8 (19)
Icteric	8 (19)
Pallor	3 (7)
Seizure	1 (2)
Local findings	
Enlargement	25 (58)
Erythema	25 (58)
Tenderness	17 (40)
Fluctuance	13 (30)
Skin abrasion	9 (21)
Spontaneous drainage	9 (21)
Blisters	7 (16)
Fracture	1 (4)
Systemic findings	
Fever^d^	28 (65)
Leucocytosis^e^	19 (44)
Irritability	11 (26)
Icterus	10 (23)
Meningitis	11 (26)
Surgical intervention	
Aspiration	2 (5)
Incision and drainage	13 (30)
Aspiration followed by incision and drainage	18 (42)
Aspiration followed by debridement and evacuation	7 (16)
Additional bone re-movement	5 (12)
None^f^	3 (7)
Complications	
Osteomyelitis	18 (42)
Sinus venous thrombosis	1 (2)
Outcome	
Full recovery	35 (81)
Death	2 (5)
Hydrocephalus	1 (2)
No follow-up	5 (12)

^a^Gestational age ≤ 37 weeks

^b^ ≥ 18 h before delivery

^c^Urinary samples taken in *n* = 28 (60 %)

^d^Body temperature ≥ 38 °C, in *n* = 6 patients the temperature was not documented

^e^Total white blood cell count ≥ 15 × 10^9^/l, in *n* = 7 patients the white blood count was not documented

^f^ *n* = 2 died before an intervention was possible

Local findings indicating infection were secondary enlargement (*n* = 25, 58 %), erythema (*n* = 25, 58 %), tenderness (*n* = 17, 40 %), fluctuance (*n* = 13, 30 %) or skin abrasions (*n* = 9, 21 %). Systemic findings included fever (*n* = 28, 65 %), leucocytosis (*n* = 19, 44 %) or irritability (*n* = 11, 26 %). The white blood cell count was available from 23 patients, the median was 18 (range 5.5–34.5, mean 17) × 10^9^/l. The C-reactive protein levels were available from 14 infants, the median was 166 (range 40–280, mean 168) mg/l.

Twenty-six percent (*n* = 11) of infants were diagnosed with meningitis. The median age at time of diagnosis was 17 (range 7–98, mean 23) days, which is seven days later than the median age at presentation. Patients presenting with sepsis and meningitis were diagnosed at a younger median age than children with osteomyelitis at 12, 18 and 22 days, respectively.

The main associations with infection of cephalhaematomas were instrumental delivery assistance in 44 % (*n* = 19) (forceps in 21 % (*n* = 9), vacuum-extraction in 21 % (*n* = 9) and both methods in one child) and sepsis in 42 % (*n* = 18). Further associated factors included the use of a scalp electrodes during delivery in 23 % (*n* = 10), skin abrasions in 21 % (*n* = 9) and prolonged rupture of membranes for more than 18 h in 7 % (*n* = 3) of infants. Maternal sepsis was not reported in any of the reviewed cases. Maternal administration of antibiotics prior or during delivery was not specified in the reviewed cases.

The used imaging methods and results are summarised in (Table 2). Osteomyelitis occurred in 38 % (*n* = 18) of infants. In eight patients the diagnosis was made because of osteolytic lesions on plain skull radiographs and in ten patients because of lytic bone changes on computed tomography images. In three patients a probable abscess was diagnosed with imaging (two via ultrasound and one via magnetic resonance imaging).

**Table 2:** Used imaging technics and results of the infants with infected cephalhaemtomas

Used imaging technic	Number of patients (% of patients with the same imaging technic)
Ultrasound	10
Probable abscess	2 (20)
Only haematoma	8 (80)
X-ray	26
Osteolytic lesions	9 (35)
Periosteal elevation	3 (12)
Fracture	1 (4)
Normal apart from haematoma	14 (54)
Computed tomography	17
Lytic bone changes	10 (59)
Normal apart from haematoma	7 (41)
Magnet resonance imaging	5
Probable abscess	1 (20)
Sinus venous thrombosis	1 (20)
Normal apart from haematoma	3 (60)

The pathogens isolated from the cephalhaematomas are summarised in (Table 3). *E. coli* was isolated from 67 % (*n* = 29) of haematomas, followed by other bacteria in much lower numbers. In patients without trauma *E. coli* was isolated in 75 % (*n* = 12/16), while in patients with skin abrasions it was isolated in 44 % (*n* = 4/9). In all of the 18 infants with sepsis, Gram negative rods were isolated from blood cultures, 17 of them were identified as *E. coli*. In all of the 11 infants with meningitis, Gram negative rods were isolated from cerebrospinal fluid; eight of them were *E. coli*. Only in 1 of the 28 children, who had a urine analysis, a urinary tract infection was found. However, while the pathogen isolated from urine was enterococcus, a culture from the cephalhaematoma of the same child grew *E. coli*. One child did not have an aspirate of the cephalhaematoma, but because the infant was found to have *E. coli* bacteraemia, meningitis and osteomyelitis of the parietal bone as well as skin lesions above the cephalhaematoma, it was presumed that the cephalhaematoma was infected.

**Table 3:** Pathogens isolated from infected cephalhaematomas

Bacteria	Number (%)
*Escherichia coli (E. coli)*	29 (67)
*Bacillus proteus (B. proteus)*	2 (5)
*Gardnerella vaginalis (G. vaginalis)*	2 (5)
*Escherichia hermanii (E. hermanii)*	1 (2)
*Streptococcus pneumoniae (S. pneumoniae)*	1 (2)
Beta-hemolytic streptococci	1 (2)
*Staphylococcus epidermidis (S. epidermidis)*	1 (2)
*Paracolobactrum coliforme*	1 (2)
*Bacteroides*	1 (2)
Gram negative rods	1 (2)
≥ 2 species	2 (5)
*Staphylococcus aureus, Streptococcus agalactiae and*	1 (2)
*Peptostreptococcus assaharolyticsus*	
*Escherichia coli and anaerobic streptococci*	1 (2)
Total	43
Clinical condition	Number of patients (% of patients with the same condition)
Skin abrasion	9
*E. coli*	4 (44)
*G. vaginalis*	2 (22)
*E. coli and Streptococci*	1 (11)
*S. epidermidis*	1 (11)
*P. coliforme*	1 (11)
Osteomyelitis	18
*E. coli*	12 (67)
*E. hermanii*	1 (6)
*S. pneumoniae*	1 (6)
*S. epidermidis*	1 (6)
Beta-hemolytic streptococci	1 (6)
*G. vaginalis*	1 (6)
*P. coliforme*	1 (6)
Vacuum-/forceps deliveries	19
*E. coli*	14 (74)
≥ 2 species	2 (11)
*Bacteroides*	1 (5)
*G. vaginalis*	1 (5)
*P. coliforme*	1 (5)
Sepsis	18
*E. coli*	17 (94)
Gram negative rods	1 (6)
Meningitis	11
*E. coli*	9 (82)
*E. hermanii*	1 (9)
Gram negative rods	1 (9)
Non-traumatic^a^	16
*E. coli*	12 (75)
*B. proteus*	2 (13)
Gram negative rods	1 (6)
Beta-hemolytic streptococci	1 (6)

^a^spontanous delivery without the use of a scalp electrode and no skin abrasion

All children received antibiotic treatment. The median duration was 21 (range 5–67, mean 27, data available from *n* = 41) days. The choice of antibiotics and the duration of therapy were heterogeneous. All but one infant were initially treated with intravenous antibiotics. Eleven patients were switched to oral antibiotics after a median duration of 21 (range 4–46, mean 19) days.

Surgical management was most commonly aspiration followed by incision and drainage (42 %, *n* = 18) or incision and drainage only (30 %, *n* = 13). In two infants aspiration was the only surgical intervention. Scalp bone was partially removed in 12 % (*n* = 5) of infants. Two patients died before a surgical intervention was possible and one child healed without a surgical intervention.

The median duration of hospitalisation was 23 days (range 5–47, mean 38, data available from *n* = 38). Six children were hospitalised twice. Of the two children who died, one suffered from unmanageable sepsis and meningitis, while the other one was found death in his bed 13 days after being treated for *E. coli* meningitis. Post-mortem examination revealed a grossly infected cephalhaematoma with growth of *E. coli* from an aspirate.

An infected cephalhaematoma is a rare, but potentially life-threating condition. Half of the infants with infected cephalhaemtoma present with non-specific signs of sepsis, such as fever, reduced feeding or lethargy and the other half because of changes of the haematoma, most frequently secondary enlargement or erythema of the overlying skin. Sepsis, instrumental assistance during delivery, the use of scalp electrodes and skin abrasions are the most important associated risk factors. Plain radiographs, computed tomography or magnetic resonance imaging have limited power to determine if a cephalhaematoma is infected, but can help in identifying associated osteomyelitis. Inflammatory markers in blood are often elevated, but this does not necessary mean that there is an infection. In two infants an infection of a cephalhaemtoma was suspected because of raised inflammatory markers, but no organisms were isolated from the haematomas or various other body fluids [2, 38]. At our clinic, we also looked after an infant who presented with a cephalhaemtoma and remarkably elevated inflammatory markers. The infant healed without any antibiotics or other interventions. Therefore, we suggest that elevated inflammatory markers can be part of the reabsorption process of haematomas and do not necessarily indicate that there is an infection, which can cause further difficulties in diagnosis.

Although, aspiration is contraindicated as a treatment option in cephalhaematomas, because of the potential risk of inoculating microbes, a percutaneous tap is necessary for the diagnosis of an infection.

Overall, *E. coli* is by far the most frequent pathogen responsible for infecting cephalhaematomas. Given that the identified associated risk factors for infection of cephalhaematomas are skin abrasions, instrumental assistance and the use of scalp electrodes during delivery, it is not surprising that entry for organisms acquired from the birth canal is facilitated. However, *E. coli* is more frequently isolated in non-traumatic cases than it is in patients with skin lesions. A further interesting finding is that even though skin abrasions are a risk factor for infection, no case of infection with *Staphylococcus aureus* as causative organism was reported.

Apart from primary invasion of pathogens, secondary infection associated with sepsis or meningitis is a further pathogenetic mechanism. Here, the spectrum of microbes is expected to be the same than in neonates with sepsis and/or meningitis without cephalhaematomas. However, studies including similar case numbers of neonates with sepsis or meningitis with comparable demographic and clinical characteristics (community-acquired late onset sepsis in term babies from developed countries before the implementation of intrapartum antibiotic prophylaxis) identified much lower numbers of Gram negative rods and *E. coli* as causative pathogens [39]. The proportion of *E. coli* in isolated late-onset meningitis is also much lower (32 %) than in infants with infected cephalhaematomas and meningitis (82 %) [40]. A further interesting finding is the rarity of Group B streptococci (GBS) as causative organisms in infected cephalhaematomas. Since the peripartal antibiotic prophylaxis for GBS does not influence the incidence of late-onset sepsis and most children with infected cephalhaematomas present at an age of 3 weeks or more, one would expect higher rates of GBS.

When an infant with a cephalhaematoma shows a decline in general well-being, fever or local signs of inflammation, infection of the haematoma should be suspected. Parents need to be informed about the potential risk of infection in cephalhaematomas when leaving the birth clinic.

Antibiotic treatment should cover the typical causative organism of neonatal sepsis/meningitis, particularly *E. coli*. Surgical intervention might be necessary.

## References

[1] Kao HC, Huang YC, Lin TY (1999). Infected cephalohematoma associated with sepsis and skull osteomyelitis: report of one case. Am J Perinatol.

[2] Paul SP, Goodman A (2011). Potential complications of neonatal cephalhaematoma in the community: when to refer to the paediatric team?. J Fam Health Care.

[3] Wong CS, Cheah FC (2012). Cephalhematoma infected by Escherichia coli presenting as an extensive scalp abscess. J Pediatr Surg.

[4] Nakwan N, Wannaro J, Dissaneevate P, Kritsaneepaiboon S, Chokephaibulkit K (2011). Septicemia, meningitis, and skull osteomyelitis complicating infected cephalhematoma caused by ESBL-producing Escherichia coli. Southeast Asian J Trop Med Public Health.

[5] Pollack S, Kassis I, Soudack M, Sprecher H, Sujov P, Guilburd JN, Makhoul IR (2007). Infected subgaleal hematoma in a neonate. Pediatr Infect Dis J.

[6] Eggink BH, Richardson CJ, Rowen JL (2004). Gardnerella vaginalis-infected scalp hematoma associated with electronic fetal monitoring. Pediatr Infect Dis J.

[7] Dahl KM, Barry J, DeBiasi RL (2002). Escherichia hermannii infection of a cephalohematoma: case report, review of the literature, and description of a novel invasive pathogen. Clin Infect Dis.

[8] Goodwin MD, Persing JA, Duncan CC, Shin JH (2000). Spontaneously infected cephalohematoma: case report and review of the literature. J Craniofac Surg.

[9] LeBlanc CM, Allen UD, Ventureyra E (1995). Cephalhematomas revisited. When should a diagnostic tap be performed?. Clin Pediatr.

[10] Lee PY (1990). Infected cephalhaematoma and neonatal osteomyelitis. J Infect.

[11] Lee Y, Berg RB (1971). Cephalhematoma infected with Bacteroides. Am J Dis Child.

[12] Mohon RT, Mehalic TF, Grimes CK, Philip AG (1986). Infected cephalhematoma and neonatal osteomyelitis of the skull. Pediatr Infect Dis.

[13] Hegde HR (1980). Infected cephalhematoma associated with placement of scalp electrode. Can Med Assoc J.

[14] Tan KL (1972). Infected cephalhaematoma. Aust Paediatr J.

[15] Levy HL, O’Connor JF, Ingall D (1967). Bacteremia, infected cephalhematoma, and osteomyelitis of the skull in a newborn. Am J Dis Child.

[16] Chen MH, Yang JC, Huang JS (2006). MRI features of an infected cephalhaematoma in a neonate. J Clin Neurosci.

[17] Chan MS, Wong YC, Lau SP, Lau KY, Ou Y (2002). MRI and CT findings of infected cephalhaematoma complicated by skull vault osteomyelitis, transverse venous sinus thrombosis and cerebellar haemorrhage. Pediatr Radiol.

[18] Nishi J, Kaji T, Tokuda K, Shinkoda Y, Okawa T, Noguchi H, Takamatsu H, Yoshinaga M (2005). A case of adrenal and cephalhematoma abscesses caused by Escherichia coli after forceps delivery. Pediatr Int.

[19] Nightingale LM, Eaton CB, Fruehan AE, Waldman JB, Clark WB, Lepow ML (1986). Cephalhematoma complicated by osteomyelitis presumed due to Gardnerella vaginalis. JAMA.

[20] Ellis SS, Montgomery JR, Wagner M, Hill RM (1974). Osteomyelitis complicating neonatal cephalhematoma. Am J Dis Child.

[21] Jacobson M, Lander HB, Spiegel IJ (1960). Spontaneous infection of cephalhematoma with recovery. J Pediatr.

[22] Weiss KJ, Edwards MS, Hay LM, Allen CH (2009). Escherichia coli--infected cephalohematoma in an infant. Clin Pediatr.

[23] Fan HC, Hua YM, Juan CJ, Fang YM, Cheng SN, Wang CC (2002). Infected cephalohematoma associated with sepsis and scalp cellulitis: a case report. J Microbiol Immunol Infect.

[24] Listinsky JL, Wood BP, Ekholm SE (1986). Parietal osteomyelitis and epidural abscess: a delayed complication of fetal monitoring. Pediatr Radiol.

[25] Kersten CM, Moellering CM, Mato S (2008). Spontaneous drainage of neonatal cephalohematoma: a delayed complication of scalp abscess. Clin Pediatr.

[26] Blom NA, Vreede WB (1993). Infected cephalhematomas associated with osteomyelitis, sepsis and meningitis. Pediatr Infect Dis J.

[27] Gordon HS, Aronow J (1955). Escherichia coli meningitis in five-day-old infant; report of a case with complete recovery. J Am Med Assoc.

[28] Burry VF, Hellerstein S (1966). Septicemia and subperiosteal cepbalbematomas. J Pediatr.

[29] Overturf GD, Balfour G (1975). Osteomyelitis and sepsis: severe complications of fetal monitoring. Pediatrics.

[30] Miedema CJ, Ruige M, Kimpen JL (1999). Primarily infected cephalhematoma and osteomyelitis in a newborn. Eur J Med Res.

[31] Paul SP, Taylor TM, Edate S (2009). Cephalhaematoma – a benign condition with serious complications: case report and literature review. Infant.

[32] Chan MC, Boon WH (1972). Infected cephalhaematoma. J Singapore Paediatr Soc.

[33] Huang CS, Cheng KJ, Huang CB (2002). Infected cephalohematoma complicated with meningitis: report of one case. Acta Paediatr Taiwan.

[34] Handrick W, Huckel D, Spencker FB, Kuhnert C (1983). Infected scalp hematoma. Zentralbl Gynakol.

[35] Meignier M, Renaud P, Robert R, Roze JC, Rigal E, Mouzard A (1989). Cephalhematoma infection in neonatal septicemia. Pediatrie.

[36] Sarlangue J, Cavert MH, Pedespan L, Demarquez JL (1996). Spontaneous Escherichia coli infection of cephalhematoma. Annales De Pediatrie.

[37] Van Helleputte C, Dupont V, Barthels S, Aeby A (2010). Escherichia coli meningitis and parietal osteomyelitis in an infant: a rare complication of cephalohematoma. Rev Med Brux.

[38] Latuff H (1963). Spontaneous infection of cephalhematoma in the newborn infant. (Presentation of 2 Cases). Rev Obstet Ginecol Venez.

[39] Greenberg D, Shinwell ES, Yagupsky P, Greenberg S, Leibovitz E, Mazor M, Dagan R (1997). A prospective study of neonatal sepsis and meningitis in southern Israel. Pediatr Infect Dis J.

[40] Gaschignard J, Levy C, Bingen E, Cohen R (2012). Epidemiology of Escherichia coli neonatal meningitis. Arch Pediatr.

